# Static Postural Stability in Women during and after Pregnancy: A Prospective Longitudinal Study

**DOI:** 10.1371/journal.pone.0124207

**Published:** 2015-06-08

**Authors:** Agnieszka Opala-Berdzik, Janusz W. Błaszczyk, Bogdan Bacik, Joanna Cieślińska-Świder, Dariusz Świder, Grzegorz Sobota, Andrzej Markiewicz

**Affiliations:** 1 Department of Physiotherapy in Internal Diseases, Academy of Physical Education, Katowice, Poland; 2 Department of Human Motor Behavior, Academy of Physical Education, Katowice, Poland; 3 Nencki Institute of Experimental Biology, Polish Academy of Sciences, Warsaw, Poland; 4 Department of Physiotherapy of the Nervous and Locomotor Systems, Academy of Physical Education, Katowice, Poland; 5 Institute of Computer Science, Silesian University of Technology, Gliwice, Poland; West Virginia University, UNITED STATES

## Abstract

This longitudinal study aimed to compare static postural stability in women between early pregnancy, advanced pregnancy, and at 2 and 6 months postpartum. Forty-five pregnant women were enrolled and 31 completed the protocol. Data were collected at 7–16 and 34–39 weeks gestation, and at 6–10 and 26–30 weeks postpartum. For each subject, the center of foot pressure path length and mean velocity (with directional subcomponents) were computed from 30-s long quiet-standing trials on a stationary force plate with eyes open or closed. The body mass, stance width, and sleep duration within 24 h before testing were also recorded. Static postural stability was not different between pregnancy and postpartum, except for the anterior posterior sway tested in the eyes-closed condition, which was significantly increased in late pregnancy compared to that at 2 and 6 months postpartum. Pregnant/postpartum women’s body mass weakly positively correlated with anterior-posterior sway in the eyes-closed condition and their stance width weakly positively correlated with the anterior-posterior sway in the eyes-open condition. No effect of sleep duration on postural sway was found. Our findings indicate that under visual deprivation conditions women in advanced pregnancy may have decreased static stability compared to their non-pregnant state.

## Introduction

The fall incidence for pregnant women is estimated to be 27% [1], similar to that of people aged 65 years and over (30%) [2]. Based on the literature, one in four pregnant women falls and one in ten falls two or more times during the gestation period [1]. Falls during pregnancy may result in injury to the mother, including fractures, contusions, and sprains, as well as in an increased risk of preterm labor, placental abruption, fetal distress, and fetal hypoxia [3,4]. Good postural control is fundamental for balance maintenance and recovery. In static force-plate posturography, an increased sway of the human body during quiet standing may indicate a decline in postural stability. An unstable posture may prevent optimal motor activity and be the cause of accidental falls [5,6].

Gestational weight gain and its asymmetrical distribution mainly in the anterior abdominal region [7], adaptive postural changes necessary for the anterior-posterior center of gravity location readjustment [8,9], as well as increased joint laxity [10,11] may lead to the changes in a pregnant woman’s static stability. The transient stability changes may also be present in the postpartum period due to increased connective tissue laxity [12] and altered posture [13]. Lateral stability, however, may be preserved in pregnancy because of the adaptive increase in stance width [14]. Pregnant and postpartum women often report that they do not get adequate sleep, and sleep deprivation may also affect postural stability [15].

Several posturographic studies suggested alterations in postural stability during pregnancy and postpartum [14,16–20]. Four previous studies indicated a decline in static postural stability with progression of pregnancy, but some of the detailed findings regarding postural sway measures are contradictory [14,16–18]. One study reported decreased static stability also at 6–8 weeks postpartum [16]. Although the impact of body mass and stance width on static stability in pregnancy was considered in previous studies, the data are inconsistent and insufficient [14,16,17]. Only the study by Butler et al. [16] of 12 pregnant/postpartum women evaluated the relation of body mass with postural sway and no association between these factors was detected. Two other studies examined stance width in 15 [14] and 20 [17] women during the perinatal period. Jang et al. [14] reported an increased stance width with progression of pregnancy and its strong association with postural sway, whereas Oliveira et al. [17] reported no changes in this measure during pregnancy. None of the studies to date evaluated the relationship between the amount of sleep and postural sway characteristics during pregnancy and postpartum.

The present study aimed to compare static postural stability in healthy women between early pregnancy, advanced pregnancy, and at 2 and 6 months postpartum. We hypothesized that static stability is altered in advanced pregnancy and at 2 months after delivery. Our secondary aim was to investigate whether body mass, base of support width, and amount of sleep within 24 h before testing might affect static stability of pregnant/postpartum women.

## Materials and Methods

This study was conducted following the approval of the Senate Ethics Committee of the Katowice Academy of Physical Education, Poland.

Forty-five healthy pregnant women were enrolled in the study. The women were directed to the testing by obstetricians from antenatal clinics in the region of Upper Silesia, Poland. All were singleton gestations and were included in the study if enrolled by their 16^th^ week of pregnancy. Exclusion criteria for pregnant women were any conditions considered by the obstetrician to indicate a high-risk pregnancy. None of the women included in the study had a history of any musculoskeletal or neurologic abnormalities, uncorrectable vision disorders, obesity, diabetes mellitus, or any other medical conditions that affect postural stability. Subjects were excluded if they were under treatment with any medication that would affect their balance. Eligibility criteria were confirmed by physical examination and a medical interview.

Women enrolled in the study reported for testing to the Biomechanics Laboratory at the Department of Human Motor Behavior at the Academy of Physical Education in Katowice. The aim of the study and experimental procedures were explained to all subjects and written informed consent was obtained.

The women were tested on four occasions: two visits were scheduled during pregnancy (at 16 weeks or earlier and again ~3 weeks before the due date) and then two follow-up visits after delivery (~2 and 6 months postpartum). Results from the last session were treated as a reference for the remaining three sessions.

Fourteen women were unable to participate in one (n = 9) or two (n = 5) of the sessions. The reasons for their absences were: complications related to pregnancy (n = 6), delivery prior to scheduled visit in advanced pregnancy (n = 3), problems with scheduling childcare for the time of the visit (n = 5), and relocation (n = 5). Data for those women were excluded from the study. Therefore, 31 women (26 primigravida and 5 multigravida) participated in all four test sessions. At the start of testing, the mean ± standard deviation (SD) age of the women was 28.2 ± 3.6 years (range 20–38 years) and mean ± SD height was 165.5 ± 5.3 cm. The subjects’ characteristics at the four data collection sessions are presented in Table 1.

**Table 1:** Characteristics of 31 women in early (P1) and advanced (P2) pregnancy and at 2 (P3) and 6 (P4) months postpartum.*

	**Gestation** (weeks)	**BMI** (kg/m^2^)	**Body mass** (kg)	**Stance width** (mm)	**Sleep length** (h)
**P1**	13.1±2.5	21.9±2.5	60.3±8.9	277±5.8	8.6±1.7
**P2**	36.2±1.2	26.5±2.9	72.8±10.3	299±29.7	7.5±1.2
	**Postpartum** (weeks)				
**P3**	7.8±1.4	23.0±2.7	63.2±9.6	276±28.9	6.6±1.5
**P4**	26.1±0.7	22.2±2.6	61.0±9.4	280±27.2	6.8±1.8

* Data are shown as means ± SD. BMI–body mass index

At the beginning of each session, the pregnant women were asked to complete a survey that included questions regarding any medication intake, musculoskeletal complaints and amount of sleep within 24 h before testing. Subject height was recorded at the initial visit and body mass was recorded at each visit. The BMI was calculated after each data collection.

Posturographic tests were performed at each session. The women were instructed to be barefoot and stand quietly with their arms at their sides and at a comfortable stance on a stable force plate (model 9281C, Kistler Instruments Corp, Winterthur, Switzerland), and tests were performed according to the manufacturer’s operating instructions. Recording started 10 s after the woman assumed the quiet standing position on the platform. Two 30-s trials were recorded first with the eyes open (looking straight ahead at a wall 3 m away) and next with the eyes closed. Short rest breaks, up to 1 min, separated the trials to avoid any discomfort.

A pregnant/postpartum subject’s preferred stance width in a comfortable standing position was recorded immediately after completion of the last trial by measuring the distance between the lateral margins of the feet at the most distant points in the frontal plane.

The center of pressure (COP) signals transmitted from the force plate were amplified and sampled at 100 samples/s. They were digitally filtered with a 12^th^ order low-pass Chebychev type II filter at a 7-Hz cut-off frequency. The COP path length and mean velocity with their directional (anterior–posterior and medial–lateral) subcomponents were calculated using Matlab (Mathworks, Natick, MA USA). The measures were computed on the basis of the means of two trials for the eyes-open condition and two trials for the eyes-closed condition [21].

The COP is defined as the point of the concentration of the pressure of the body over the soles of the feet that accommodates a spontaneous postural sway of the body in an upright stance. COP movements reflect both the center of gravity excursions and reaction forces due to muscular activity [6]. The COP path length is the distance the COP travels over the time of a trial. Mean velocity is a ratio of the path length to the trial period. Both sway indices are considered very reliable and particularly valuable in clinical practice [21–26].

Statistical analyses were performed on the COP measures recorded in the eyes-open and eyes-closed conditions, as well as on body mass, base of support width and sleep duration 24 h before testing. A repeated-measures analysis of variance (RM ANOVA) was performed to determine the effects of the four periods (early and late pregnancy, 2 and 6 months postpartum) on a given measure. When the results of the ANOVA were significant, Fisher's least significant difference (LSD) post hoc test was applied to analyze differences between means for particular sessions. For measures failing to meet the assumptions for the RM ANOVA (stance width, sleep length within 24 h before testing, medial-lateral COP path length and velocity in eyes closed conditions), a Friedman ANOVA was performed. Spearman’s Rank Correlation Coefficient was used to test whether the pregnant/postpartum subject’s body mass, base of support width, and amount of sleep 24 h before testing were correlated with the COP measures. The level of significance was set at p≤0.05. Analyses were performed using the Statistica 9.0 (StatSoft Inc., Tulsa, OK USA).

## Results

From the 30-s long quiet-standing trials in the eyes-open and eyes-closed conditions, COP path length and mean velocity (with directional subcomponents) were compared in women between early pregnancy, advanced pregnancy, and at 2 months and 6 months postpartum. Pregnant women’s COP measures did not differ significantly between the four test sessions in the eyes-open or eyes-closed conditions (p>0.05), except for the anterior-posterior path length and velocity in the eyes-closed condition, which decreased significantly from late pregnancy to 2 months postpartum (p = 0.014) and remained decreased at 6 months postpartum (p = 0.017; Table 2).

**Table 2:** Center of pressure measures reflecting spontaneous body sway during 30 s of quiet standing with the eyes open (EO) or closed (EC) in 31 women in early (P1) and advanced (P2) pregnancy and at 2 (P3) and 6 (P4) months postpartum.*

Measure	P1	P2	P3	P4
**AP path length** (mm)				
EO	142.0±32.6	148.0±40.0	141.3±30.0	143.1±31.8
EC	208.9±59.9	**224.2**±**68.6** **	**199.6**±**63.6** **	**200.5**±**65.3** **
**AP velocity** (mm/s)				
EO	4.8±1.1	5.0±1.4	4.8±1.0	4.8±1.1
EC	7.1±2.0	**7.6**±**2.3** **	**6.8**±**2.2** **	**6.8**±**2.2** **
**ML path length** (mm)				
EO	230.4±49.6	217.1±49.4	227.3±49.5	228.0±42.5
EC	297.7±78.1	279.7±75.2	284.7±84.5	298.0±99.0
**ML velocity** (mm/s)				
EO	7.8±1.7	7.4±1.7	7.7±1.7	7.7±1.4
EC	10.1±2.7	9.5±2.6	9.7±2.9	10.1±3.4
**Total path length** (mm)				
EO	292.2 ± 62.3	287.5±69.6	291.4±64.8	292.2±59.5
EC	391.8±103.5	393.7±108.1	379.1±112.2	392.1±126.9
**Total velocity** (mm/s)				
EO	9.9±2.1	9.7±2.4	9.9±2.2	9.9±2.0
EC	13.3±3.5	13.3±3.7	12.8±7.7	13.3±4.3

AP–anterior-posterior plane, ML–medial-lateral plane.

* Data are shown as means ± SD.

** Significantly decreased from P2 to P3 (p = 0.014) and from P2 to P4 (p = 0.017), Fisher’s LSD post-hoc; RM ANOVA: F_3, 90_ = 2.71, p = 0.049.

The pregnant women’s body mass, stance width, and amount of sleep 24 h before testing were compared between the four sessions and the association of these factors with the COP measures was assessed. Body mass increased significantly from early to late pregnancy (p<0.0001), decreased from late pregnancy to 2 months after delivery (p<0.0001), and further decreased from 2 to 6 months post-birth (p = 0.001). At 2 months after delivery, body mass was significantly higher than that in early pregnancy (p<0.0001). Body mass did not differ between early pregnancy and 6 months postpartum (p>0.05). Non-significant correlations were found between the body mass and COP measures (p>0.05), except for a significant weak positive correlation with the COP anterior-posterior path length and mean velocity in the eyes-closed condition (r = 0.206, p<0.05).

The base of support width increased significantly from early to late pregnancy (p<0.0001), decreased from late pregnancy to 2 months postpartum (p<0.0001), and remained decreased at 6 months after delivery (p = 0.0001). The stance width did not correlate significantly with the COP measures (p>0.05), except for a significant weak positive correlation with the COP anterior-posterior path length and velocity in the eyes-open condition (r = 0.199, p<0.05).

The amount of sleep 24 h before testing reported by the women ranged from 6 to 13 h in early pregnancy, 3 to 10 h in advanced pregnancy, 4 to 10 h at 2 months, and 2 to 12 at 6 months postpartum. On average, women slept significantly fewer hours in late pregnancy than in early pregnancy (p = 0.005). In addition, they slept significantly fewer hours at 2 and 6 months postpartum compared to early pregnancy (p<0.0001). At 2 months after delivery, the women reported significantly less sleep than in advanced pregnancy (p = 0.024). No statistically significant correlation was detected between the COP measures and the amount of sleep 24 h before testing (p>0.05).

## Discussion

The primary objective of this longitudinal study was to compare static postural stability between early pregnancy, advanced pregnancy, and at 2 and 6 months postpartum. We expected different postural sway characteristics in women during advanced pregnancy and at 2 months postpartum. The results of the present study, however, indicated that only the anterior-posterior sway in the eyes-closed conditions was increased in advanced pregnancy and it was recovered at 2 months after delivery. This finding suggests that women in advanced pregnancy, when deprived of visual cues, have a decline in anterior-posterior static stability.

From a biomechanical perspective, the body of a pregnant woman undergoes pronounced changes in the sagittal plane. The increasing weight in the anterior abdominal region leads to an increased forward-flexing torque of the lumbar spine motion segments and ankle joints. To reduce energy expenditure in terms of muscle work and to preserve anterior-posterior stability while standing, a pregnant woman adapts her posture by assuming a slight posterior body tilt [9,27]. According to Whitcome et al. [8], the increased lumbar lordosis with the progression of pregnancy permits mothers to maintain a stable, unchanged anterior-posterior position of the center of mass. This may explain why under normal visual conditions, our findings demonstrated unchanged anterior-posterior static stability in women during the perinatal period. Jang et al. [14], however, reported that the anterior-posterior postural sway tended to increase over the second through third trimesters of pregnancy, and significantly decreased postpartum compared to that during advanced pregnancy.

In the present study, anterior-posterior sway tended to increase in the eyes-closed condition from early to advanced pregnancy, and significantly decreased from advanced pregnancy to 2 and 6 months postpartum. This finding suggests that the full adaptation of a pregnant woman’s postural control observed under normal visual conditions is diminished when the central nervous system is challenged because of the lack of visual cues. While visual stimuli may help pregnant women to maintain an optimal anterior-posterior posture alignment in the standing position, this possibility was not investigated in the present study. Increased reliance on visual cues with the progression of pregnancy was the conclusion of the studies by Butler et al. [16] and Oliveira et al. [17]. Dunning et al. [1] reported that poor lighting and an obstructed view accounted for 17.1% and 10.7% of falls in pregnancy. The findings from these studies together with our findings support the notion that women in advancing pregnancy may be at increased risk of falling when their vision is compromised.

Our findings indicate that lateral stability is preserved in women throughout the perinatal period regardless of visual conditions. The reason for this may be that a pregnant woman’s body shape changes evenly in the frontal plane, and the increasing mass is more equally distributed compared to that in the sagittal plane. Our findings are partially consistent with those of other studies. Jang et al. [14] reported that lateral sway measures, which were unchanged during pregnancy, increased after delivery. Nagai et al. [18] observed an unchanged lateral sway in women in their third trimester of pregnancy during standing with the eyes open, which was decreased under visual deprivation conditions compared with the controls.

We examined whether there was any association of a pregnant/postpartum woman’s body mass, stance width, and amount of sleep 24 h before testing with static postural stability. Our findings indicated no association except for weak positive correlations of the body mass and stance width with the anterior-posterior postural sway. The main factors that are expected to influence postural control in pregnancy are increased joint laxity [10,11] and unevenly distributed increased body mass [7]. At the same time, the adaptive changes of an anterior-posterior posture alignment [8] and stance width [14] are observed. It may be that these different factors acting together attenuate any impact of body mass or stance width on the postural sway in healthy pregnant women. Similarly, Butler at al. [16] found no correlation between the change in weight in pregnancy and postpartum and the change in the postural sway measures. Jang et al. [14], however, reported a strong positive correlation between stance width and anterior-posterior sway, as well as a strong negative correlation between stance width and lateral sway during pregnancy and postpartum.

Thus, our results indicate that static postural stability under normal visual conditions does not change throughout the perinatal period. This finding suggests that in healthy women, the postural control system of the central nervous system, receiving inputs from visual, vestibular, and somatosensory sources, adapts to the changes that occur in pregnancy and postpartum. In the eyes closed condition, however, postural sway is increased in advanced pregnancy. Based on this finding and those of other studies [1,16,17], health care providers should educate pregnant women regarding the possible increased risk of falling under compromised vision conditions, such as darkness or obstructed view. Pregnant women may also benefit from specific balance exercises offered in their prenatal exercise classes.

The findings of the present study are based on a sample size twice as large as that in similar studies [14,16], but the size is still relatively small and the results should be considered with caution. Fourteen women from our pregnant group were not able to complete one or two of the four scheduled test sessions. Their data were excluded to assure a stronger statistical analysis and to present clear demographics of the group. This markedly reduced the number of study participants.

Further studies are needed to investigate the possible association of joint laxity and posture changes with the postural sway measures of women in the perinatal period. Also, evaluation of dynamic stability in the eyes-open and eyes-closed conditions would provide more insight into the postural stability characteristics during pregnancy and postpartum. An assessment of the effect of specific exercise programs on postural stability in pregnancy would also be warranted.

## Supporting Information

S1 Dataset Study dataset. (XLS)
